# Acceptance of Disability: Determinants of Overcoming Social Frustration

**DOI:** 10.5539/gjhs.v7n3p317

**Published:** 2015-01-25

**Authors:** Elena Valeryevna Morozova, Svetlana Vasilyevna Shmeleva, Elena Aleksandrovna Sorokoumova, Vera Borisovna Nikishina, Larisa Vasilyevna Abdalina

**Affiliations:** 1 Russian State Social University, 4,1, V. Pika, Moscow, 129226, Russia

**Keywords:** internal picture of disability, personality, status “disabled”, adaptation, rehabilitation, psycho-correction

## Abstract

The article is devoted to the subjective reaction of patients at different stages of disabling disease, in the context of the formation of a specific cognitive-emotional and motivational model of “internal picture of disability”, depending on the severity of social frustration as the most important deconditioning factor. We wanted to identify psychological determinant of the specificity of adaptive activity of the patient to the situation disabling disease, depending on the level of increase social frustration. Nature of adaptation to the disabling disease depending on the level of increase social frustration expressed by: 1) decrease in self-esteem of patient self-efficacy with an increase in subjective experience of disability; 2) the growing tension of personal protective mechanisms; 3) reductions coping competence, which, depending on the rise of frustration, becomes effective instead of the rational-intelligent, more maladaptive emotional.

## 1. Introduction

### 1.1 Introduce the Problem

Consequence of potentially disabling disease may be the situation is actually established person of a group of disability. The potentially disabling disease might have as a consequence the actual assessment of a particular group of disability to a person. According to current regulatory criteria to establish disability, its actual availability stipulates restriction due to the appearance in some spheres of life, but the reaction to this fact may be at least ambiguous: accepting of the restriction with regrets for the missed opportunities (just hoping to obtain a “disability group”) or accepting the fact of restriction with looking for various opportunities and options for self-realization (without refusing from medical care) (Bonkalo, 2013).

For persons at various stages of disabling disease there is a topical issue of personal definition through a commitment to rehabilitation (restoration of the socio-economic and socio-psychological status), or the formation of the passive adaptive behavior, which often reaches the degree of social dependency. The importance of this problem is due to a significant number of persons with disabilities resulting from various disabling diseases, injuries, defects, who do not have the assessed disability (or have lost it), but eager its reestablishment (return), despite of the existing normative criteria and classifications for establishing of such status in the Russian Federation.

As a result of the development of medical rehabilitation technologies makes it possible for many patients suffering from a number of disabling diseases to improve or restore their physiological functions, which according to the regulatory criteria means the partial or full rehabilitation, and therefore the change of a social status. The paradox is that the victories gained by medicine in the sector of human’s rehabilitation, would start the socio-psychological mechanisms of formation of disadaptive accomodational strategies of an individual.

Modern expert approaches imply that a person due to disease or injury with an illness or injury resulting in minor disabilities will be able to return and be integrated into the society, providing that the existing violations would not affect their fundamental living environment (mobility, communication, self-care, work, education, self-management behavior and orientation in the surrounding reality). However, some people with minor functional disabilities and therefore unrecognized invalids who are confident in their social failure identify themselves as disabled, categorically unwilling to be rehabilitated, and as the only possibility of adapting regard the status of “disabled.”

The greater problem present the cases when disability group is reduced as a result of a successful rehabilitation (e.g. changes from 2 to 3) or lost at all, through the successful rehabilitation, therefore depriving a person of the corresponding socio-economic support. Such socio-economic devaluation of position of the patient is a serious psychological problem due to the inability to accept the change in the social status and return to an active life without the usual patterns of socio-economic benefits, as evidenced by numerous disagreements with the expert solutions declared by people who have not been assessed disabled, as well as by those ones whose have had this status reduced or withdrawn.

### 1.2 Specification of Problem

Thus, the rehabilitation creates a paradoxical formation of a disadaptive psychological condition of personality. This condition is caused by the improvement of physiological functioning together with the drastic change of the socio-economic context due to the failure to obtain, or loss of a “disabled” status, which leads a person to the disadaptive condition of psychosocial frustration because of the loss of benefits and compensations. Such situations need to be studied for the purpose of ensuring effective strategies for rehabilitation of this category of citizens and prevention of mental disability of the person experiencing the frustration expressed by the social background of instability in the social status.

Study of patients at the stage adaptation of invalidity, as well as adaptation and coping with illness (coping behavior) (Tim, Andrew, & Charlotte, 2005; Munro, Lewin, & Swart, 2007; Byrne, Walsh, & Murphy, 2005; Kissane, 2004; Shah, Dunlay, & Ting, 2009; Mellon, Northouse, & Weiss, 2006) for various disabling pathologies subject of numerous studies (Roy-Byrne, Davidson, Kessler et al., 2008; Tang, Patao, & Chuang, 2013; Cunningham, 2004; Denollet, Pedersen, Ong, Erdman, Serruys, & van Domburg, 2006; Bauer, Caro, Beach et al., 2012; Evers, Kraaimaat, & van Lankveld, 2001; Garcia & Mann, 2003; Godin & Kok, 1996; Heim, 1995; Morisky, Green, & Levine, 1986; Prochaska & DiClemente, 1992; Prochaska & Velicer, 1997; Rosenstock, Strecher, Becker, 1988; Rozema, Völlink, Lechner, 2009; Weinstein, 1993; Williams, Ehde, Smith, Czerniecki, Hoffman, & Robinson, 2004). However, practical experience of experts on disability shows the need for study)of the phenomenon of subjective responses of patients who are at various stages of disability, including not only its initial establishment, but also the stage when a disability as a result of the effective partial rehabilitation decreases or as a result of full rehabilitation is lost at all).

A number of researches are dedicated to the study of specifics of various types of rehabilitation and adaptation of patients with different disabling pathologies.

However, the practical experts’ experience on the assessment of disability proves the necessity of the study of this phenomenon of subjective reactions of patients in connection with the possible status dynamics of social category “disabled”.

## 2. Method

To identify the specificity of personal adaptation at various stages of disabling disease, the following psychodiagnostic methods were used: Diagnosis of the level of social frustration” (USF) (St. Petersburg Research Institute. Bekhterev) (Wasserman, 2004); H. Khaimah`s methods to identify the dominant strategy of coping behavior, test “Lifestyle index” (Nabibullina & Tuhtarova, 2003), Scale E. Heim, St. Petersburg Research Institute. VM Spondylitis); a modified version of the method “scales” (Dembo-Rubinstein) modified for the purpose of the study - option of self-esteem.

One of the objectives of the study of internal picture of disability as a personal reaction of patients who are in conditions of disabling disease, was to study the psychological characteristics of persons applying for disability, its gain or return (n=460), and of patients with the actual presence of similar disabling diseases, but avoiding the actual (legal) documentation of this social status (n=180).

In two study groups there were found statistically significant differences in the total index level of social frustration subjects, which is a leading deconditioning factor (p<0.01). Both groups are not uniform in the severity of the reaction, which has allowed reformulating the question of private study as follows: what are the main changes in the dynamics of adaptive reactions with an increase in the level of social frustration?

To answer this question, the sample was divided into three equal size groups: those with a low level of social frustration, with average values and, finally, with the high level of social frustration.

## 3. The Results

The results of the test voltage of mechanisms for psychological defense (Table 1), allows us speaking about the rise of performance of most types for psychological defense in connection with the increase in the level of social frustration (except for “denials” and “intellectualization”).

**Table 1:** Severity of psychological defense reactions in three groups of subjects with different levels of social frustration

No	groups			

	mechanisms for psychological defense	1 group with a low level of social frustration	2 group with average values	3 group with the high level of social frustration
1.	2.	3.	4.	5.
2.	denials	5,14	5,59	5,75
3.	suppression	3,93	4,93	6,15
4.	regression	4,69	7,16	8,84
5.	compensation	3,66	4,70	5,13
6.	projection	7,95	8,97	10,33
7.	replacing	3,16	4,17	5,38
8.	intellectualization	5,66	5,71	5,75
9.	reactive formations	3,19	4,68	5,44

Quantitative assessment of the severity of differences has showed that statistically significant differences (p <0.05) are both between groups 1 and 2 and between groups 2 and 3 in terms of protective responses, like “suppression”, “regression”, “projection”, “replacing”, “reactive formations”. In terms of “compensation” such differences were found only between the 1st and 3rd groups. Uniquely these results evidenced in favor of the need massive psychological, psycho-support people in a difficult social situation disabling disease with possible change of status “disabled”. Such assistance will be significantly reduced the severity of protective mechanisms opening the way for the conscious choice of exit strategies from the frustrating situation of social adaptation crisis.

Analysis of the results of the assessment of dominance of certain coping strategies in the studied groups is showed a significant dominance of cognitive coping strategies in all three groups (Table 2). However, at low severity of social frustration severity of cognitive strategies in the first group is statistically significantly exceeded (p <0.001) and the frequency of their use in the second group and the third one. A similar index but less pronounced (p <0.01), and is pertained to total rates of behavioral coping strategies.

**Table 2:** The ratio of expression of the three major classes of coping strategies (the comparison of the total mortality in the three groups of subjects)

	Behavioral coping strategies	Cognitive coping strategy	Emotional coping strategy

1.	2.	3.	4.
group patients with a low level of social frustration	16,71	20,83	8,39
group patients with average values of social frustration	12,64	15,54	9,83
group patients with the high level of social frustration	14,04	16,63	15,28

Increase in severity is not unnoticed subjects in the matter of choice of emotional coping strategy. In the third group, they become the most prominent and have a statistically significant predominance (p<0.001) on the indicators and the first group and the second one.

As a result of data comparison for study of coping strategies and reactions of psychological defense it was found out that the growth of social frustration is accompanied by a decrease of the relative role of cognitive coping mechanisms and an increase of emotional mechanisms. The dominant type of reaction within the growing social frustration is shifted from cognitive sphere to the emotional one, from coping strategies to reactions of psychological protection.

In our concept leading role of “internal picture of disability” exceptional value is belonged to the human self-determination, in the context of its own assessment of self-efficacy that is determined the importance of consideration indicators of self-examination with the inclusion of this method positions of disability evaluation, which in accordance with the existing regulatory approaches – the disability is established.

The results of our research have evidenced the decisive factor of reactions to social frustration is self-identification with the status of “disabled.

As we are developing the concept of the leading role of “internal picture of disability” exceptional value belongs to the self-determination rights, in the context of assessment of self-efficacy, hence the importance of self-examination performance with the inclusion of this technique positions Disability evaluation, which, in accordance with existing regulatory approaches established disability (Table 3) (Morozova, 2008; Morozova & Shmeleva, 2011).

**Table 3:** Intensity of quantitative indicators of self-esteem in the three groups of subjects, depending on the level of increase social frustration

self-assessment scale patient’s individual psychological characteristics and self-efficacy in the main spheres of life	1 group with a low level of social frustration	2 group with average of social frustration values	3 group with the high level of social frustration

1.	2.	3.	4.
health	50,90	47,96	33,99
mind	74,34	62,96	45,52
character	74,58	60,56	54,68
happiness	78,55	56,71	33,61
appearance	65,05	50,18	42,28
ability to move	77,53	76,52	51,39
ability to self	83,25	66,91	50,89
ability to control the behavior	81,27	66,97	53,92
employability	72,76	46,18	33,61
ability to communicate	85,28	66,78	54,37
ability to learn	75,06	44,74	36,19
self-assessment of the level of disability	33,19	55,67	79,75

There is an important fact that statistically significant differences (p <0.001) are recorded on all indicators of self-esteem in comparison with the first and second groups. With an increase in social frustration only two indicators are remained unchanged: “strength of character” and “the ability to learn» (p> 0.05).

## 4. Discussion

Thus comparing the three groups of subjects with disabling disease and varying degrees of social frustration there is found that the increase the level of frustration, firstly, there is prepared by a sharp decline in the self-assessment of all the main parameters of the self-attitude(except for the admission of “disabled” - this index has an inverse dynamics) secondly, accompanied by increased severity of reactions “psychological defense”, reducing the adaptive human capabilities(in particular, forming a circle of neurotic reactions in man’s relation to the surrounding reality), thirdly, reducing the volume and repertoire of coping responses, changing their structure in favor of less effective coping mechanisms of emotional response. Implementation of coping behavior is possible by coping strategies on the basis of personal and environmental coping resources, and is the result of a complex interaction. In both cases, the coping behavior aimed at the implementation and achievement of adaptation or readjustment of the individual to the new prevailing conditions, it is extremely important for the person in the process of adapting to the debilitating disease. Adequate subjective perception of disability allows a person to form an adaptive problem-critical behavior aimed at redefining the understanding and acceptance of themselves in the new conditions of life, which in fact is a mechanism for adaptation. While a distorted perception of the situation disabling disease, promotes maladaptive behavior associated with the departure of the solution of problems in the implementation of adaptation to their changing capabilities, and affects the whole adaptation process as a whole. Frustration linked with such personal qualities of the person, such as high intelligence and self-confidence, independence, self-confidence and a positive attitude toward self and others, which makes it possible to overcome frustrating situations in life more productive and more likely even tempers, indicating the possibility of the individual to self-actualization.

## 5. Conclusion

One of the impotent problems of the social policy of the state to its citizens is the prevention of disability, including psychological aspects associated with disability. Investigation of the internal picture of disability of persons with different levels of social frustration has revealed significant personal phenomena that allow to establish the fact of personal transformation on the basic parameters of self-awareness and coping in difficult situations disabling disease. Analysis of the identified psychological options surveyed patients with different levels of social frustration, reveals characteristic personality phenomena that allow to establish the fact of personal transformation on the basic parameters of self-awareness, formation of personal protective response in the form of the rise of a distorted perception of reality, the implementation of maladaptive coping behavior to the difficult situation of the disease with an increase in level of social frustration in the major social and interpersonal spheres of life. The results obtained revealed the target rehabilitation dispositions important for applied research, which may be used and should be all the professionals involved in the rehabilitation process (psychologists, therapists, specialists in social work, etc.). Obtained results could be an important element in the preparation of individual rehabilitation programs of remedial work with patients at various stages of disabling disease (before the actual onset of disability, in period of decline and loss). This study opens up the prospect for further study of psychological adaptation and rehabilitation, in fact non-disabled, but with conviction, claiming the present social status, that in case of successful remedial work with the person to avoid maladaptive strategies “psychological disability.” If successful, timely remedial work with the person to adopt situational changes as a result of the loss of status “disabled”, reduction of disability or its failure to identify, perhaps to balance the psychological state of the person, to help him avoid maladaptive strategies of social adaptation in the form of “psychological disability” stigma and unfounded identification with invalid community with virtually absent to this objective of health and social indicators.

It should be noted that the results of empirical research conducted may be theoretical and methodological basis for the development of effective programs for persons with persistent disturbances in health.
